# Spring buds of European woody plants have old ^14^C age

**DOI:** 10.1016/j.heliyon.2024.e32777

**Published:** 2024-06-10

**Authors:** Tamás Varga, Dominik Nagy, Mihály Molnár, A.J. Timothy Jull, István Futó, Zsuzsa Lisztes-Szabó

**Affiliations:** a International Radiocarbon AMS Competence and Training (INTERACT) Center, HUN-REN Institute for Nuclear Research, Debrecen, H-4026, Hungary; b Isotoptech Ltd., Debrecen, H-4026, Hungary; c Department of Geosciences, University of Arizona, Tucson, AZ, 85721, USA; d University of Arizona AMS Laboratory, Tucson, AZ, 85721, USA; e HUN-REN, Institute for Nuclear Research, Debrecen, H-4026, Hungary; f Department of Botany, University of Debrecen, H-4032, Hungary

**Keywords:** Bomb-radiocarbon, Woody plant, Carbon pool, Reserved carbon mobilization, Bud

## Abstract

Trees and shrubs maintain carbon reserves to support their functions during periods when metabolic demand exceeds carbon supply, such as during the dormant season. To gain a better understanding of carbon storage and utilisation dynamics of eight woody plant species in temperate Central Europe, bud scale and leaf samples were collected to determine the radiocarbon age of fresh sprouts in trees and shrubs, at three background sites avoiding local emissions that may influence affect the observed ^14^C/^12^C ratio. The accelerator mass spectrometry-based bomb-radiocarbon approach, to determine the age of the mobilized carbon in the plant bud samples from storage, was complemented by stable carbon isotope measurements. The bomb-radiocarbon dating technique was used to determine the age of the samples, while a northern hemispheric atmosphere ^14^CO_2_ dataset was used to calibrate the radiocarbon ages of the plant samples. The youngest observed calibrated radiocarbon age of the buds was over 4 years, and the oldest was even 9 years old. There was no significant difference between the ages of bud scales and embryonal leaf laminas. Our results show that there is a considerable amount of stored older carbon in the woody stems that can be used to produce buds in spring, which is a complex mixture of stored carbon of different ages, but there is no relationship between the radiocarbon age and the stable carbon isotope composition. The observed results show that not only the tree species, but shrubs also can store and use significantly older carbon pools, the carbon storage intensity is similar for trees with trunks and short-stemmed shrubs branching directly above the ground, i.e. carbon storage starts in young twigs and continues in ageing branches.

## 1 Introduction

There are nutrient and carbon pools in plants that can be effectively used during the photosynthetically inactive periods to support the nutrient needs of plants during germination or sprouting. Photosynthesis is carried out by a type of ground tissue called the assimilatory parenchyma or chlorenchyma. This is not yet present in the perennial woody plants during the dormant season (before green leaves develop in spring), but the plant needs energy, that can be supplied from these pools (mainly starch, sucrose, glucose, fructose, lipids). These carbon pools are commonly referred to in the literature as nonstructural carbon (NSC), but these sources do not only support the carbon demand but can also act as a nitrogen pool [1]. These NSCcan help through active and passive processes and can support in the response to stressors [[1], [2], [3]]. Different NSC forms are present in different proportions in individuals of different plant species, but the high proportion of starch is the most characteristic [[4], [5], [6]]. Starch differs from free sugars in that it is osmotically inactive and accumulates as a storage compound during periods of carbon surplus [1].

Some targeted studies have been published on this topic, mainly focusing on economically or ecologically important species or regions such as maple trees in the USA or the Amazon region, but the temperate, overwintering woody species of the East Central Europe have not been investigated from this point of view of ^14^C in individual trees and species in temperate forests, and from the point of view the potential covariation of ^13^C and ^14^C [[7], [8], [9]]. Here, we focus on the role of old carbon in bud production in a temperate climate. Woody plants shoots remain in an embryonic state covered and protected by bud scales throughout the winter and develop into revived sprouts in the spring. The fresh buds continue to grow until the end of summer and woody plants then enter a dormancy period until the following spring. Thus, embryonic tissues covered with non-photosynthesizing scales necessarily develop from reserve nutrients (NSC) and are suitable for examining the average age of allocated NSC.

Various methods can be used to investigate the NSC pools in plant liquids, emitted CO_2_ through stems, fresh buds and sprouts. These plant parts and products are active when the photosynthesis and assimilation are less active or completely inactive. Previous studies have shown that even new roots, buds or flowers can be produced using 2-years-old NSC in red maple trees [7]. According to Muhr et al. [8], mature trees can store and mobilise NSC that is 7–14 years old. While the photosynthetically-active period is well-defined in temperate plants during the growing season, the utilisation of stored NSC pools occurs during and outside of this period. Therefore, determining the composition of the NSC pools is complex due to the mixture of old and fresh materials, and the distribution of NSC in the plants is presumably not homogeneous. Richardson et al. [10] found that the distribution of NSC in temperate trees is not uniform. The NSC pool in older tree rings, where the mixing of NSC is stronger, was found to be older than in younger tree rings.

Radiocarbon dating combined with the atmospheric radiocarbon bomb-spike signal is a unique analytical tool for investigating the age distribution of NSC in plants [[7], [8], [9],^11^]. The carbon stored in plant carbohydrates represents the atmospheric isotopic composition at the time of allocation reflecting the ^14^C/^12^C isotopic ratio of atmospheric CO2. This is due to the plants assimilating CO2 through photosynthesis. The atmospheric bomb-spike provides a unique calibration opportunity. The atmospheric radiocarbon concentration and ^14^C/^12^C ratio almost doubled due to nuclear bomb tests in the 1950s–60s. However, after the Nuclear Test Ban Treaty (1963), this concentration rapidly decreased because of the rapid exchange of CO_2_ between the atmosphere and ocean. This caused a continuous decreasing trend in the isotopic ratio. Although the yearly decrease is not as strong as in the 70s, recent materials with calibrated ages still provide 2-3-year precision [12,13]. This approach has been used in various studies related to forensic, oceanography, atmospheric science, biosphere and plant research to investigate circulations, and turnover times, and determine precisely the age of different materials in nature. Previous NSC-related studies have also utilized this approach to estimate the turnover time of the allocated NSC in the plants [[7], [8], [9],^14^].

Radiocarbon-based studies of nonstructural carbon in trees offer valuable insights into carbon allocation within forests, aiding our understanding of the global carbon cycle. Investigating nonstructural carbon, which includes sugars and starches in leaves, branches, and roots, allows researchers to unravel how trees store and utilise carbon over time. Accurately modelling carbon fluxes in forests, including uptake during photosynthesis and release through respiration and decomposition, requires a critical understanding. Studies such as these also assist in evaluating the impact of climate change on carbon allocation patterns within trees, which inform predictions of future forest dynamics [2,10,14]. Changes in carbon allocation resulting from climate change can significantly impact the global carbon cycle, potentially creating feedback loops that could either exacerbate or alleviate climate change. For instance, alterations in carbon allocation towards roots can affect carbon storage in soil, while increased respiration during stress conditions may lead to in carbon losses from forests [15]. Therefore, it is essential to understand these mechanisms to develop effective carbon management strategies and mitigate the impacts of climate change. Nonstructural carbon studies based on radiocarbon provide a detailed understanding of how trees store, use, and release carbon, contributing to our broader understanding of the complex dynamics of the global carbon cycle and its response to environmental change.

This study reports on the use of a particular approach to determine the age of 14 temperate deciduous woody species buds at three background sampling points. The buds were formed during the early spring of 2023, when the photosynthesis was inactive due to the missing chlorenchyma. The study provides new data on woody plant carbon storage in the Carpathian Basin, where similar research has not been conducted previously. Additionally, radiocarbon dating of buds was conducted using samples of the same species from different sites, as well as individual plants of the same species from the same site. This provides further evidence to support the originality of the research.

## 2 Materials and methods

### 2.1 Samples and preparing buds

**Fig. 1:**
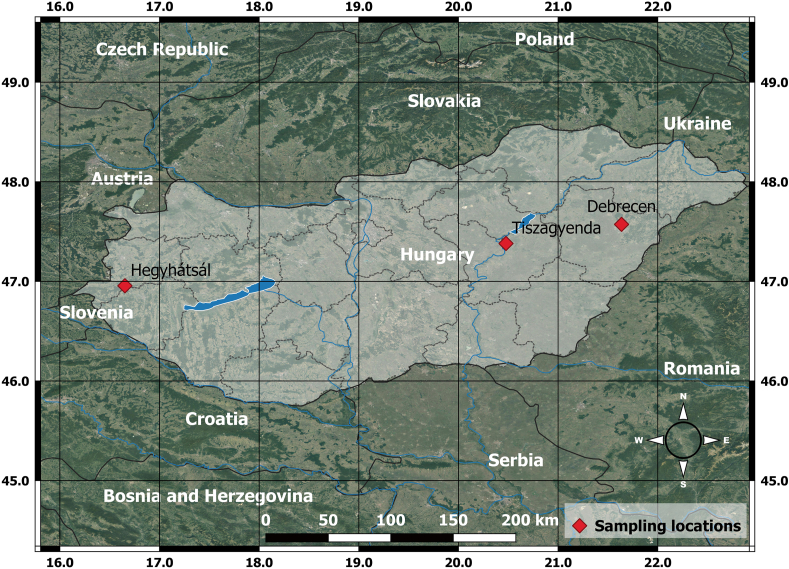
Map of the sampling sites in Hungary. The red square symbols show the location of the sampling points with the name of the nearest urban area. (For interpretation of the references to colour in this figure legend, the reader is referred to the Web version of this article.)

**Fig. 2:**
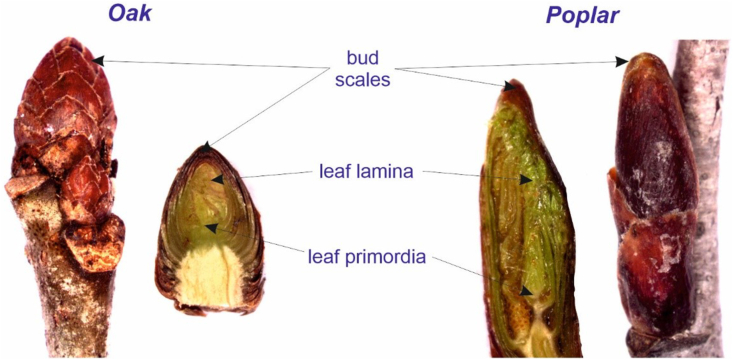
Morphology of oak (*Quercus robur*) and poplar (*Populus* sp.) trees buds. In the experiment, bud scales and leaf primordia were treated separately.

The samples were collected from Hungarian background sites (Table 1), including at the location of an atmospheric background monitoring station (Hegyhátsál). These areas are less influenced by human activity and lack nearby urban emission, such as local fossil or nuclear emissions which could affect the ^14^C/^12^C ratio of the plant samples. As the plants collect the carbon (CO_2_) through the photosynthesis, any local fossil CO_2_ emissions could impact the ^14^C/^12^C ratio of plant samples. To avoid this, we selected sampling locations in rural sites. With the exception of the Debrecen city sampling location, samples were collected from less densely forested areas surrounded by farmlands during early spring, before the buds break when there were no leaves on the plants. All of the samples were collected from mature trees or shrubs. From the trees, specifically from the youngest part of the growing branches at a height of approximately 3 m height. In case of shrubs, also the youngest part of the growing branches was chosen. Fig. 1 shows the location of the sampled trees and shrubs, while Fig. 2 displays the separated parts of plant samples used in our study.

**Table 1:** Description of the plant samples.

Sampling sites	Sampling date	Sample	Scientific name	Common name	Type
Hegyhátsál	March 02, 2023	H1	*Populus* L.	Poplar	Tree
Hegyhátsál	March 02, 2023	H2	*Cornus sanguinea* L.	Common dogwood	Shrub
Hegyhátsál	March 02, 2023	H3	*Euonymus europaeus* L.	European spindle	Shrub
Hegyhátsál	March 02, 2023	H4	*Euonymus europaeus* L.	European spindle	Shrub
Hegyhátsál	March 02, 2023	H5	*Salix* L.	Willow	Tree
Hegyhátsál	March 02, 2023	H6	*Euonymus europaeus* L.	European spindle	Shrub
Hegyhátsál	March 02, 2023	H7	*Sambucus nigra* L.	European elder	Shrub
Hegyhátsál	March 02, 2023	H8	*Picea pungens Engelm.*	Blue spruce	Tree
Tiszagyenda	March 04, 2023	T1	*Acer platanoides* L.	Norway maple	Tree
Tiszagyenda	March 04, 2023	T2	*Populus* L.	Poplar	Tree
Tiszagyenda	March 04, 2023	T4	*Salix* L.	Willow	Tree
Tiszagyenda	March 04, 2023	T5	*Populus* L.	Poplar	Tree
Debrecen, Great Forest	March 19, 2022	D1	*Quercus robur* L.	Pedunculate oak	Tree

Differences in the soil properties and precipitation levels exist among the sampling points. In 2023, the precipitation amount at Hegyhátsál was approximately 600 mm, while at Debrecen and Tiszagyenda it was approximately 450 mm and below 400 mm, respectively. The soil types at the three sites are Luvisoil (Hegyhátsl), Phaeozem (Tiszagyenda) and Arenosol (Debrecen) according to the World Reference Base for Soil Resources. These differences may affect plant growth across the three sampling sites.

As samples, we collected woody twigs that were a maximum of 2-3-year-old and did not contain photosynthetic bark tissue The study involved separating the youngest apical buds from the twig ends under a dissecting microscope, and then each bud was divided into two parts. One sub-sample consisted of bud scales without green tissues, the other sub-sample consisted the leaves lamina and leaf primordia with green tissue that were still under differentiation (hereafter referred to as leaves) and they were still enclosed in bud scales without sunlight. (see Fig. 2). No chemical preparation was applied at this stage.

### 2.2 Carbon isotope measurements

The individual separated samples were chemically prepared to remove any contaminants from sampling and laboratory procedures. The standard, internationally applied ABA radiocarbon chemical preparation was used in the INTERACT AMS laboratory, where the samples were heated in a heating block at 70 °C, and then the samples were sequentially washed with 6 ml of 1 N HCl to remove the dust and carbonates, then 4 % NaOH solution to remove humic acids, and other contaminants such as alcohols, phenols and carboxyl groups. Finally, the samples were washed again with 1 N HCl, respectively. Between and after the HCl and NaOH wash steps, the samples were rinsed with ultrapure water until a neutral pH was achieved [16]. These steps effectively clean the samples and remove contaminants without affecting the ^14^C/^12^C and ^13^C/^12^C ratios of the samples or introducing significant carbon contamination. Following the chemical preparation, the samples were dried in a heating block at 50 °C until a constant mass was achieved. Prior to combustion, the samples were stored in a dark refrigerator at −20 °C to prevent any biological activity.

For combustion, about 2 mg samples were placed in glass tubes with 300 mg of MnO_2_ powder added as an oxidant. The tubes were then sealed under vacuum (<5*10^−2^ mbar) to prevent atmospheric CO_2_ contamination The sealed tubes were heated and the samples were combusted in a muffle furnace at 550 °C for 12 h. The released CO_2_ from the organic material underwent purification in a dedicated vacuum line, where the H_2_O was removed by trapping it with an isopropyl alcohol–dry ice mixture at approximately-78 °C, while CO_2_ was selectively trapped by a liquid nitrogen trap at −196 °C. For further information on the system used and its performance with organic material, refer to Janovics et al. [17]. The remaining negligible amount of non-carbonaceous gases do not interfere the measurement.

Half of the trapped CO_2_ gas was separated for δ^13^C measurement and the other half was separated for graphitization and AMS ^14^C/^12^C measurement. To measure δ^13^C we used a Thermo Finnigan Delta PLUS XP Isotope Ratio Mass Spectrometer was used. The stable carbon isotope ratio of samples was compared to reference materials to avoid systematic errors. The δ^13^C values were calculated using the following equation (Equation (1)):

(1) δC=1000Rsample−RreferenceRReference13

where R_sample_ and R_reference_ are the measured ^13^C/^12^C isotope ratio of the sample and isotope ratio of the reference material, respectively. The δ^13^C values are expressed against the Vienna Pee Dee Belemnite (VPDB) reference.

The CO_2_ was purified and then graphitized using the sealed tube graphitization method with Zn, TiH, and iron powder reagents and catalysts. A stepped-heating protocol was employed, starting at 500 °C for 3 h, followed by 550 °C for 5 h [18]. The resulting solid graphite samples were analysed for their ^14^C/^12^C ratio using the accelerator mass spectrometer at the INTERACT laboratory, in Debrecen, Hungary [19]. In this study, the results are presented as “pMC” (percent modern carbon, where “modern” is defined as 1950CE, or 95 % of the ^14^C/^12^C ratio of the NIST oxalic-I standard [20,21]. The calibration of radiocarbon ages was performed using the OxCal 4.4 online software was using the F^14^C radiocarbon units [12,[22], [23], [24]]. The OxCal 4.4 online software uses monthly mean ^14^CO_2_ data from Hua et al. [12] as a calibration curve for different zones. We used the Bomb21NH1 zone, as it fully covers Hungary until 2019. The calibration software does not assume any seasonality of ^14^C fixation. Due to the seasonal fluctuations, and the declining trend of the specific atmospheric ^14^CO_2_, the calibrated age results could span multiple years instead of a single exact date. In evaluating the calibrated age intervals and comparing them to the date of sample collection (as expected year), we used the youngest age to avoid overinterpretation.

## 3 Results and discussion– the freshly sprouted plant buds are older than 3 years

**Fig. 3:**
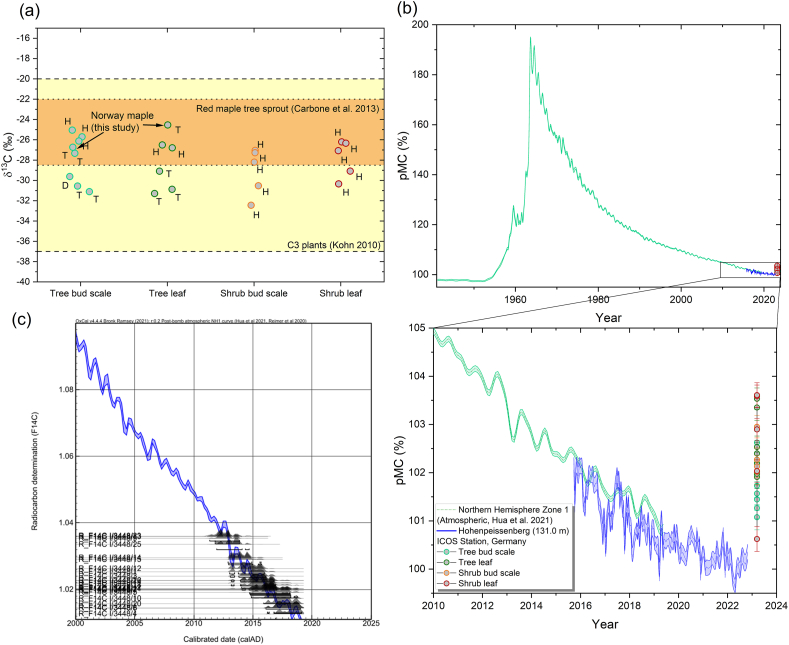
Stable carbon isotope data of plant samples(a), where the yellow colour band shows the typical δ^13^C isotope ratio range of C3 plants and orange shows the red maple tree sprout δ^13^C range from Carbone et al. [7], plot of raw radiocarbon data with reference atmospheric values (b) and figure of calibrated ages using OxCal online software (c). The letters (T, D, H) denote the sampling sites as Tiszagyenda (T), Debrecen (D), Hegyhátsál (H). (For interpretation of the references to colour in this figure legend, the reader is referred to the Web version of this article.)

**Fig. 4:**
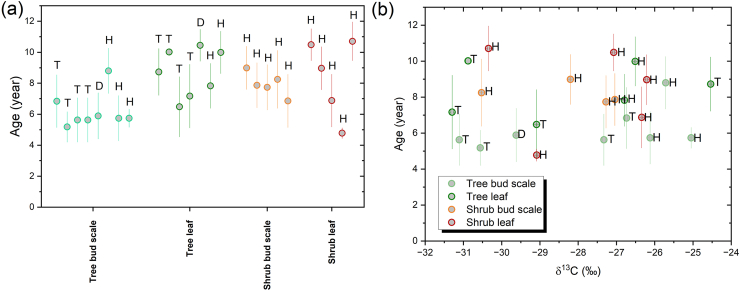
Plot of mean radiocarbon ages of plant samples (a) and scatter plot of mean radiocarbon ages versus δ^13^C results of plant materials (b). The letters (T, D, H) denote the sampling sites as Tiszagyenda (T), Debrecen (D), Hegyhátsál (H).

The results of the δ^13^C analysis do not show any significant differences and fall within the expected wider range for terrestrial C3 plants, ranging from −32,45 ‰ to −24,54 ‰ (VPDB). There are no significant differences in the δ^13^C signature between the bud scale and leaf samples (Fig. 3a), and there is no significant difference between the bud scale and leaf sample from the same plant. The radiocarbon and stable isotope ratio results are available in the Supplementary material file S1. As demonstrated by Carbone et al. [7], sprouts can exhibit a broad range of δ^13^C values, but do not represent a distinct δ^13^C signature. However, the red maple sprouts show a slightly higher values, which overlap with our results. The results of the European Norway maple, as observed in this study, fall completely within the wide range of results observed by Carbone et al. [7]. This wide range of δ^13^C patterns can presumably be attributed to the complex biological production of plant materials and the complex, inhomogeneous structure of the stored carbon pool within the plants. Based on these results, the δ^13^C values alone are insufficient to properly support the carbon source studies of bud samples adequately. The results overlap within different groups, and cover too wide a range. There is no correlation between the observed δ^13^C values and the corresponding radiocarbon ages or values (see Fig. 4b). This suggests that there is no effect, such as aging, that could impact the δ^13^C through fractionation during biochemical processes. Storage pools likely mix fresh photosynthates with carbon stored over longer periods and operationally extracted pools can represent a range of chemical compounds with different biochemical δ^13^C signatures. The δ^13^C signature is affected by meteorological parameters, the age of the tree, and other physiological and environmental parameters over the years. Muhr et al. [8] also did not find any correlation between the radiocarbon age of fresh maple syrup and the corresponding δ^13^C values, which is consistent with our findings. Carbone et al. [7] found a connection between the radiocarbon age of stemwood sugar and the δ^13^C values. However, they also did not observe any correlation between the radiocarbon age of sprouts and the δ^13^C values, which is also consistent with our work.

The radiocarbon AMS ^1^ results indicate higher ^4^C/^12^C ratio than the last available atmospheric ^14^CO_2_ measurement (middle of 2022), with a value of 100.4 ± 0.2 % pMC value from the German background atmospheric ICOS station, Hohenpeissenberg (Fig. 3b). As the measurement precision is about 0.2–0.3 %, the difference in several cases is higher than 3 σ error. The recent atmospheric value presumably is likely to be even lower than this level, due to the decline of the atmospheric ^14^C bomb-peak. Our previous research has shown the agreement between local atmospheric ^14^CO_2_ and Jungfraujoch ^14^CO_2_ during the vegetation period [25]. Therefore, the shrub and tree buds have significant older carbon contributions, which are assimilated in the previous years, even several years before the sampling. The lowest values are 101.1 ± 0.3 and 100.6 ± 0.3 %, the highest are 103.5 ± 0.2 and 103.6 ± 0.3 in the case of trees and shrubs. The average values for trees and shrubs are 102.1 ± 0.7 (n = 15) and 102.4 ± 0.9 (n = 10), which are within 3 σ error of each other. Therefore, these groups have similar ^14^C/^12^C ratios. It is worth noting that there is no significant difference between the ^14^C/^12^C ratios of bud scales and leaves.

This study presents a dataset of plant bud ^14^C from Hungary and East-Central Europe, demonstrating a significant amount of stored carbon from the previous years both in both trees and shrubs. The finding suggest that plants can mobilise stored carbon for the production of fresh materials and the synthesis of novel tissues, such as bud parts (scale leaf). This dataset could aid the understanding of stored carbon, carbon pools and carbon allocation studies in plants. The results of the ^14^C analysis indicates a significant amount of assimilated and allocated carbon from previous growing seasons. However, it is unclear whether this carbon is continuously used or stored and used after a given time period. The carbon used to produce buds is a mixture of the carbon sources, including significantly older carbon-containing sources with a significantly higher ^14^C/^12^C ratio. As the ^14^C/^12^C ratio is a bulk ratio, it may contain carbon that is much older than the observed bulk ratio and ages. We can only observe the turnover time (TT), mean radiocarbon age or average resident time of the bulk carbon pool.

The raw pMC results can be calibrated using the OxCal online calibration software. However, it is important to note that the database in this software only uses monthly clean air radiocarbon datasets until early 2019 (Fig. 3c and a) [12]. This indicates that the carbon in the buds is at least four or more years old, as the sampling was conducted in early 2023, as it could be calibrated (Fig. 3c) with this software, they have comparable radiocarbon value (^14^C/^12^C ratio) with the atmospheric radiocarbon in or before 2019. No correlation was observed between the calculated calibrated ages and measured δ^13^C values (see Fig. 4b). This indicates that there is no direct connection between the apparent calibrated ^14^C age of the stored carbon and its stable isotope signature. It is likely that many other factors affect the measured δ^13^C signature, such as the biochemical processes in the plant, meteorological conditions and plant stress factors.

The calibrated ^14^C dates indicate that the mean age of the carbon, and the turnover time in the buds are 6.0 ± 2.3 and 6.8 ± 0.9 years in the trees and shrubs, respectively. The oldest age measured for the trees was 9.9 years (*Quercus robur*) and for the shrubs, it was 9.5 years (*Sambucus nigra*), while the youngest was 4.2 and 4.4 years old in the group of trees and shrubs. Therefore, the groups of trees and shrubs can be considered to be uniform based on the studied species bud ^14^C values. (Fig. 4a). There is no significant difference in age between the studied bud scale and leaf samples within the tree and shrub groups. The results show a minimum 2-3-year wide interval (Fig. 4a), indicating this carbon pool does not have a well-defined age, but varies depending on other factors.

Comparison of the tree bud scale and leaf samples from the same plant reveals that the carbon in the bud scale is generally younger (average 2.4 years, but the SD is also 2.5 years). Only one sample was observed where the fresh tree leaf was younger than its bud scale by only one year (*Populus* sp., Hegyhátsál). Although the trend is not statistically significant, it may be due to the fact that the bud scales are usually already formed during the previous year's growing season, resulting in a higher likelihood of the younger carbon isotope ratio is being inherited by the bud scales. However, the leaves covered by the bud scales begin to develop in early springtime. Despite the lack of significance in this pattern, the opposite is true for shrub bud scale and leaf samples. The mean difference between the samples is only 0.9 years, which may be considered negligible. However, the standard deviation is also 2.5 five years, similar to that observed in the case of trees samples. Additionally, the greatest difference observed is 4.3 years, which is larger than the greatest difference observed in the case of tree samples. The preliminary results suggest that the difference between the bud scale and leaf within one plant may reflect the inhomogeneity of the NSC storage and usage, rather than any differences in plant-related metabolism. This indicates that the NSC pool storage and usage within one plant is not uniform and can be affected by various environmental and biological factors. The rate of storage and use of the assimilation starch may vary by species, and there may also be a differences in bud location within the branch system. Due to these complex conditions, the offsets in ages are random within certain limits. All shrub samples were collected from the narrow area (<0.2 km^2^) surrounding the Hungarian background atmospheric station. Most of the shrub bud samples were collected from mature European spindle plants. However, the ^14^C ages of the bud samples vary even more than 4 years. This indicates that the ^14^C is affected not only by the local meteorological and environmental factors but also by the characteristics of the plants. However, the results of the bud scale analysis for the shrub samples are not comprehensive as those for the leaf parts.

In the case of *Euonymus europaeus* L. samples, for which we have 3 different individual sampled plants, the ages ranging from 4.4 to 7.6 years. These plants were growing in close proximity to each other, and were collected from the same sampling site. Similarly, for *Populus* L. samples, the ages ranges from 4.2 to 9.9 years. As the samples were collected from different sampling sites with a distance of approximately 290 km, the local conditions may have influenced the ^14^C values in the plants (see Fig. 1). No significant differences were observed in the mean calibrated ages of plant tissues from the various sampling sites. The mean calibrated age of plants at the Tiszagyenda site is 5.6 ± 2.0 year, at the Debrecen site was 6.9 ± 3.5 years, and at the Hegyhátsál site was 6.7 ± 1.8 years.

No significant differences were found between the species. Additionally, the bud scale samples of trees showed a more uniform distribution compared to the leaf samples, with the highest and lowest bud scale and one of the lowest and highest leaf radiocarbon ages found in *Populus* L. samples from the same sampling area in Tiszagyenda. The bud scale sample of trees shows a more uniform distribution compared to the leaf samples. When examining shrub leaf samples, it was found that Euonymus europaeus L. had lower minimum radiocarbon ages compared to *Sambucus nigra* L. and *Cornus sanguinea* L. samples. The oldest *Euonymus europaeus* L., sample was 8 years old, while the *Sambucus nigra* L. and *Cornus sanguinea* L. samples were 9.5 years old. However, this difference was not observed in the shrub bud scales. Based on these results, there can be a higher difference between individual plant samples than between different species from different sampling sites.

According to our findings from the East-Central European plant study, both the trees and shrubs possess a considerable amount of older carbon pool that can be utilized for bud production. This carbon pool can be as old as 9 years, indicating a significant amount of aged carbon present in the trees that can be utilized for leaf production. The results are comparable to those of Muhr et al. [8], who found turnover times of 3–5 years or longer in xylem sap sugar samples of sugar maple (*Acer saccharum* *Marsh*) from two Canadian locations. They also observed variable turnover times, during their 1-month-long continuous sampling and measurement campaign, ranging from approximately 2 to 6 years. Carbone et al. [7] also found variable sprout (radiocarbon) ages. Stump sprouts were formed from nonstructural carbon more than 10 years old, which is older than our findings However, they also found other samples that were only 2 ± 1 years old. Muhr et al. [26] also found that Amazonian trees can remobilize stored nonstructured carbon up to 17 years old, as observed in emitted CO_2_ through stems using the same bomb-radiocarbon (^14^C) approach as we have used. In our study, we preferably used the youngest age of the calibrated radiocarbon ages, but the whole intervals generally cover 3 years. Therefore, the mean or oldest observed age in our study can be over 9 years, which is closer to that was observed by Muhr et al. [27]. In a subsequent study, Muhr et al. [9] utilized stem chambers and discovered that the emitted CO_2_ can be dated back up to 20 years in a Peruvian study. However, they also found younger CO_2_ ages ranging from 0 to 6 years, which is similar to our study. Our findings demonstrate that not only the tropical trees can store and utilise significantly older carbon, but this is also true for temperate plants in East-Central Europe. Richardson et al. [10] found that the fast NSC pool, which is less than a year old, was used for growth and metabolic activity in temperate trees. However, our results show that the NSC pool used for sprout production utilized an older carbon pool, even older than 5 years. This indicates that these plants preferentially used aged NSC for the production of new plant tissues.

In contrast to our study, Guadinski et al. [26] found that the mature temperate trees in Tennessee used fresh carbon not older than 1–2 years to produce new roots and less than 1-year-old carbon for buds. This shows a higher turnover rate and mix of relatively fresher materials compared to our recent study. Hoch et al. [5] reported that newly-developed leaves of deciduous trees become independent from NSC reserves at an early developmental stage, and thus do not deplete reserved carbohydrate storages. Cortes and Sinclair [28] also found that the starch concentration in the twigs of mature sugar maple trees (*Acer saccharum*) increased continuously during the spring months until the first buds sprouted and photosynthesis began. Hoch et al. [5] confirmed the results of previous similar studies and concluded that NSC reserves have insignificant stem dynamics, while branches show seasonal dependence. This indicates that woody plants have sufficient NSCreserves for multiple foliage changes These data support the observation that the radiocarbon age of fresh woody plant buds is more than four-year-old.

## 4 Conclusion

Photosynthesis occurs in the leaves, and the production of leaves requires a carbon pool without photosynthetic activity in springtime. It is unclear how much carbon can be mobilized in different plant species. Only a few studies have been published on this topic, which have focused on different areas and species. The atmospheric bomb-radiocarbon approach is an effective tool to identify modern, but slightly older carbon contributions, as has already been demonstrated in atmospheric, oceanic and other studies [12]. In this recent study, we used this approach and age calibration, to detect a significantly older carbon pool in trees and shrubs. This carbon pool is used for the production of bud scales and leaves in temperate species before the photosynthetically active phase in the growing season, specifically before bud break. The age range of the observed samples was between 4 and 9 years old, which is significantly different 0 years that would correspond to the date of the sample collection in spring 2023. These ages are completely comparable to previous observations made by other groups in Canadian and Amazonian studies that used the same accelerator mass spectrometry-based radiocarbon dating method. Our investigation demonstrates that shrubs, as well as treescan store and utilise carbon that is significantly older, despite having a less extensive shoot system. Our study did not reveal any species-specific differences. However, the results from the same plant species collected at different background sites were variable. The presented findings indicate that the investigated species preferentially used old carbon pools (older than 5 years) to produce new plant tissues, leaf buds before the growing season. The finding suggest that carbon can be stored extended periods not only in trees within large and old forests, such as the Amazonian region, but also in individual trees in temperate regions. However, the age of the stored carbon pool can vary from site to site and, based on our research, can differ from tree to tree or shrub to shrub even at the same sampling location. The findings suggest that woody plants consistently store and utilise carbon from a carbon pool that is not completely fresh (0 years) nor entirely old. This process is not exclusive to trees, as even shrubs demonstrate the use of storage reserves averaging nearly a decade old. There is no correlation between the δ^13^C and age of the carbon pool, indicating no preference in the utilisation of stored carbon. No observable ageing effect, such as isotope fractionation based on the biochemical cycle, was observed. The results presented here enhance the understanding of the carbon storage in trees and provides information on the carbon pools, and storage in temperate plants. Based on our results, deforestation may have a more severe effect son the atmospheric carbon dioxide level than previously thought. This is because deforestation not only eliminates the possibility of carbon storage in structural bonds for decades through plant carbon metabolism, but also eliminates sequestration in reserve carbon pools.

## CRediT authorship contribution statement

**Tamás Varga:** Writing – original draft, Visualization, Supervision, Methodology, Investigation, Formal analysis, Data curation, Conceptualization. **Dominik Nagy:** Methodology, Investigation. **Mihály Molnár:** Writing – original draft, Supervision, Resources, Methodology, Investigation, Funding acquisition, Conceptualization. **A.J. Timothy Jull:** Writing – original draft, Supervision, Formal analysis, Conceptualization. **István Futó:** Methodology, Investigation. **Zsuzsa Lisztes-Szabó:** Writing – original draft, Visualization, Supervision, Methodology, Investigation, Formal analysis, Data curation, Conceptualization.

## Declaration of competing interest

The authors declare the following financial interests/personal relationships which may be considered as potential competing interests:

Tamas Varga reports financial support was provided by State of Hungary. If there are other authors, they declare that they have no known competing financial interests or personal relationships that could have appeared to influence the work reported in this paper.
